# An Ishmaelite

**Published:** 1888-05-05

**Authors:** Leslie Keith

**Affiliations:** Author of "The Chilcotes," "Uncle Bob's Niece," "East and West," etc.


### An Ishmaelite.

By Leslie Keith,

Author of "The Chilcotes," "Uncle Bob's Niece," "East and West," etc.

Chapter V.—Arthur's Promise.

The nine months of separation and solitude over, Fred passed, with other long-sentenced prisoners, from Millbank to Dartmoor.

Once again he traversed the London streets and heard the roar of the traffic which he could not see—as a dead man come back to life might remotely listen to once familiar sounds. How often had Fred whisked in hansoms through these same streets, bent on pleasure or amusement, the sweeter because it was stolen, over which he was now driven, a criminal himself and handcuffed to another. The most wretched in the streets out there would scarcely envy his lot.

At Paddington he suffered acutely. Suppose any old acquaintance—suppose Nellie were to see him! At the thought of the young girl, who had shared all his childish troubles and often suffered for them, a desolating sense of his shame swept over him. He was dead to Nellie now, and she to him. He would never feel the touch of her soft fingers, or look into her liquid dark eyes—never again. He had by his own act violently rent his life in two, and she belonged to the ended part of it.

Arthur would have rushed up from Oxford to the station, if but for a glimpse of his cousin, had he known the hour of the prisoner's removal, and could have got his *exeat* in term time. Indeed, he would have braved this latter risk without much compunction; but he was not there.

As the forlorn little band, close guarded by warders, set out, a pitying passenger thrust some tobacco into Fred's hand. He took it humbly, with a hanging head. Who was he to refuse any gleam of kindness? He passed the gift to his comrade, who seized it greedily, and held him a fool for parting with it.

A few miles from the great establishment on the moors, the convicts were ordered to leave the omnibus and walk. Fred stumbled out stiff and weary, cramped with the long night journey; the morning was raw and chilly, but oh, how sweet the air tasted, how clean and pure! The prisoners walked three abreast, manacled wrist to wrist in unwilling fellowship; armed warders on either side, who never for a moment relaxed the vigilance of their watch. To this psss, to the companionship of the vile, from whom he shuddered in sick distaste, had he sunk. It was a melancholy procession, and yet all the depravity and sin that walked these ways that March morning could not stain the undefiled blue of the sky, or taint the subtle odours that the wind brought across the moors. The beautiful shire is not beautiful in this region; it is wild, bleak, desolate—a fit home for sinning men and women; but no landscape had ever seemed fairer in Fred's eyes. Oh, for one hour of liberty to breast those hills, to foot them an unfettered man, with an honest man's freedom to choose the life he will. Life was all settled for Fred; for four years yet to come he was to have no say or voice in the matter. For four years? For always. Should he ever, if he lived to be an old man—and he prayed that he might be spared that misery—forget the shame that branded him and set him apart as the comrade of felons? All his mental activity was absorbed in the contemplation of the present; he could not leap, as Arthur did, to a future when his degradation should be forgotten; there was no such future for him.

From the moment the prisoner passes behind the encircling walls of Dartmoor, he leaves his identity behind; he ceases to have a name, and is known by a number. He lives by routine as part of a great and complicated machinery; he eats, works, sleeps, one had almost said by word of command, and he is never for a single instant free from observation. To whatsoever gang he belongs—whether he does his appointed task as quarryman, bricklayer, carpenter, or gardener—within doors or without, he is watched. At the very first sign of an attempted flight the warder's rifle is raised and the trigger pulled. Even in chapel, where the prayers and hymns ascend, and supplications fall from lips little used to such exercises, the guard is never absent, the rifles never unloaded.

Fred took his place among that sad congregation, and mechanically repeated the familiar words, though his heart had no voice in them. A desolating, dreadful sense of shame oppressed him, yet he never dreamt of escape. He did as he was bid, dully and hopelessly. A seven-foot cell, a shelf for bedding, a hammock slung against the wall, a wooden pail, tin mug, candle, brushes, and cleaning materials to constitute his furniture—this is what he had exchanged for the luxuries of his rooms at Oxford. There was a school slate too; he looked at it with a sad smiling irony.

"I am to be put back to school," he thought; and there rushed across him a memory of the time when he had made laborious pothooks and hangers on such a slate as this at his mother's knee. Who shall say that if he sinned he was not punished too?

He learned in time all those little devices that ameliorate a convict's lot, and preserve him from being reported adversely. To jump when the bell announced a new day; to wash and clean his cell; to make his bed; to do these and all the thousand other duties expected of him passively and spiritlessly, but with outward submission, exacted all the effort of which he was capable, and left him little room for the thoughts he panted not to entertain. He drooped so visibly after a time, that, as the summer approached, he was removed from the gang of tailors which he had joined on his arrival, and was incorporated with a parti-coloured flock engaged in gardening and farm-work.

With the open-air life and exercise he regained his health, and returning physical vigour brought with it a dulled insensibility to mental pain. He no longer suffered so acutely; the days went by in a terrible monotony—a sameness that never varied. This unchanging changelessness acted like a mental soporific—a paralysis that deadened memory and blurred all the records of the past. "As I am now, so I have always been, so I shall always be." His one desire was to forget, and he succeeded in it; but perhaps that first shocked aversion from himself that had anguished him held in it a better hope of moral recovery.

Before the first year of the dreadful five had elapsed, Arthur was hastily summoned to the house in Portland Place. He left the gaieties of his first Commemoration, and rushed up to town when he received Nellie's urgent letter. Their aunt, who was all the mother they had ever known, lay dying. In those last hours her powers seemed to revive with a final flicker. It was she who bade Nellie write.

"Arthur will come, I know," she said. "He will not grudge it when he knows that I am going away, Nellie."

"He will come," said Nellie; "but, oh Aunt Helen, what shall I do without you?"

Mrs. Eastgate laid her wasted hand on the girl's bent head. "You have been a dear daughter to me, Nellie, but we have both been timid and frightened. Ah, my child, I can see it all now; if I had been braver, if I had taken courage, I should not have been lying here a desolate, broken-hearted woman. It all began long ago, when I was as young as you, Nellie." She spoke with a solemn urgency between her pauses for breath. "Promise me, my darling, if someone stronger, who has the power, should ask you to do anything against your conscience, against your heart, promise me you will not yield?"

"I promise, I promise," said Nellie, between her tears. What would she not have promised at that moment in her yearning to give comfort to the dying woman? Mrs. Eastgate looked at her with wistful love.

"Nellie, Nellie! don't let them spoil your life as they spoiled mine. Remember, weakness may become a sin. Lift me up," she said. "I hear Arthur's step. You will leave us alone. I have a charge to give him."

Arthur would not believe in the sentence of death that had been pronounced by the doctors when he went into the room. Nellie detained him a moment in the dressing-room to whisper that he must be very gentle and quiet.

"She cannot bear much, she is very weak."

But when he went up to the bedside it seemed to him as if they were all mistaken, and that she was recovering, not dying. Her cheeks had a little delicate flush, and her eyes were bright. She was half sitting up, resting against a pile of cushions, and wrapped in a white fleecy shawl; she made him think of the pretty young lady of long ago, who had looked to his childish eyes like an angel, in the white shining dress in which she went to parties.

"You are better," he said; "you will get well. You must not leave us before——"

She looked up at him and the tears came into her eyes. In his youth and strength he made her think of her own son who ought to have been with her now.

"No, Arthur," she said; "I shall not get better. I have always been a poor, weak creature, and everything has been too much for me. I have prayed that I might live to see my boy again, but it is not to be. I sent for you to make a last request, Arthur. You will listen to my dying wish? You, who have always been my younger, dearly-loved son. You will not forsake your—your elder brother? All the world will turn against him; but you, Arthur, you who loved him too, you will not forsake him?"

Even if he had shrunk from the task, could he have resisted such pleading? Her beseeching looks were fastened on him with yearning eagerness. He took her hand in his own. He held himself erect, and there was a light of steadfast purpose in his blue eyes.

"I will never forsake him," he said simply. "We will begin the world together again. I will go with him where he goes. He is my brother. I should have stuck to him anyhow, but if it comforts you to know it——"

The inartificial words came from a very sincere heart. Arthur did not know, perhaps, what he was doing when he took the weight of his cousin's soul upon his own, but the dying woman knew that she might rely on his patience and constancy.

"I shall die more easily," she said, falling back against her pillows. The effort she had made left her shaken and exhausted. "I have a message for him," she said. "I am very weak. Come near. Stoop down that I may say it in your ear."

So Arthur knelt while those tremulous, dying lips whispered their last message of love to the prodigal who was expiating his sins in exile. She sank rapidly after that deliverance of her need, as if she were done now with all earthly concerns. Just at the last the quiet house was suddenly startled by the arrival of Cousin Fanny from the country. She had not come when that other trouble befel the stricken household, but a bereavement in the family always brought Cousin Fanny to the front.

The same loud voice came sounding upstairs as it had sounded long ago, and when Nellie ran startled out of the sick-room to look over the railing, she recognised the familiar luggage being carried in. The old-fashioned trunk with the initials "F. C.," and the curious embellishments in brass-headed nails, was there, but Fanny had brought in addition a tall and strapping daughter.

"I felt that I must come to you at once," she said, marching into the library, and taking Joseph by storm. In those critical days Mr. Eastgate had the decency to refrain from going to the City. "It was really very inconvenient, but poor Helen's condition made it a duty. Dear, dear, it is very sad, but she was always weakly. I brought Ella with me for company. Ella, shake hands with Cousin Joseph."

"It was very thoughtful of you, Fanny," said Mr. Eastgate. In the disappointment of his married life he had turned with a revived appreciation of their useful virtues to his own family. "Very thoughtful," he repeated condescendingly, "and considerate."

"Mr. Coates was unwilling I should come, but I told him I could not reconcile it to my conscience if I were not here at the last. Is that you, Arthur?" she turned as the door opened. "This is your Cousin Ella. Ella, go upstairs with Arthur, and he will get you some tea."

But the young man never so much as glanced at the blushing Ella, and he did not take the hand Cousin Fanny offered him. He held his own up in warning.

"Hush!" he said. His young face was very stern. "You are wanted upstairs," he said, looking at his uncle.

Mr. Eastgate rose and followed his nephew silently. Cousin Fanny was for coming too, but Arthur waved her back imperiously.

Death had gone up the wide, velvet-carpeted staircase before them, and was hovering on the threshold of the room where Helen Eastgate lay. For days she had only been conscious at intervals, but now and then a faint murmur escaped her. When her husband went up to the bed it was his own name he heard in that wandering, changed voice. Was it "Joseph, forgive!"

His dark, stern face worked with a sudden emotion. He stooped and kissed those pale lips, and even as he did it, their last fluttering breath went out.

*(To be continued.)*

